# RhC Phenotyping, Adsorption/Elution Test, and SSP-PCR: The Combined Test for D-Elute Phenotype Screening in Thai RhD-Negative Blood Donors

**DOI:** 10.5402/2012/358316

**Published:** 2012-11-14

**Authors:** Songsak Srijinda, Chamaiporn Suwanasophon, Unchalee Visawapoka, Malinee Pongsavee

**Affiliations:** ^1^Graduate Program in Medical Technology, Faculty of Allied Health Sciences, Thammasat University, Rangsit Campus, Rangsit, Patumthani 12121, Thailand; ^2^Division of Blood Bank, Department of Pathology, Phramongkutklao Hospital, Bangkok 10400, Thailand; ^3^Division of Immunology, Department of Clinical Pathology, Army Institute of Pathology, Bangkok 10400, Thailand; ^4^Department of Biochemistry, Phramongkutklao College of Medicine, Bangkok 10400, Thailand; ^5^Department of Medical Technology, Faculty of Allied Health Sciences, Thammasat University, Rangsit Campus, Rangsit, Patumthani 12121, Thailand

## Abstract

The Rhesus (Rh) blood group is the most polymorphic human blood group and it is clinically significant in transfusion medicine. Especially, D antigen is the most important and highly immunogenic antigen. Due to anti-D, it is the cause of the hemolytic disease of the newborn and transfusion reaction. About 0.1%–0.5% of Asian people are RhD-negative, whereas in the Thai population, the RhD-negative blood type only occurs in 0.3%. Approximately 10%–30% of RhD-negative in Eastern Asian people actually were D-elute (DEL) phenotype, the very weak D antigen that cannot be detected by indirect antiglobulin test (IAT). There are many reports about anti-D immunization in RhD-negative recipients through the transfusion of red blood cells from individuals with DEL phenotype. D-elute phenotype screening in Thai RhD-negative blood donors was studied to distinguish true RhD-negative from DEL phenotype. A total of 254 Thai serologically RhD-negative blood donors were tested for RhCE phenotypes and anti-D adsorption/elution test. In addition, RhC(+) samples were tested for RHD 1227A allele by SSP-PCR technique. The RhD-negative phenotype samples consisted of 131 ccee, 4 ccEe, 1 ccEE, 101 Ccee, 16 CCee, and 1 CcEe. The 42 Ccee and 8 CCee phenotype samples were typed as DEL phenotype and 96% of DEL samples were positive for RHD 1227A allele. The incidence of RhC(+) was 46.4%, and 48 of the 118 RhC(+) samples were positive for both anti-D adsorption/elution test and SSP-PCR technique for RHD 1227A allele. The sensitivity and specificity were 96% and 100%, respectively, for RHD 1227A detection as compared with the adsorption/elution test. In conclusion, RhC(+) phenotype can combine with anti-D adsorption/elution test and RHD 1227A allele SSP-PCR technique for distinguishing true RhD-negative from DEL phenotype.

## 1. Introduction

Rhesus (Rh) antigens are acylated red cell transmembrane protein with a molecular weight of 30–32 kDa and encoded by two highly homologous genes, *RHD *and *RHCE*. Both genes are located on chromosome 1p34.3–1p36.1 and are about 30,000 base pairs apart [1]. They have opposite orientation and are highly homologous retaining more than 90% identity [2, 3]. The Rh blood group is the most polymorphic human blood group system, of high clinically significance in transfusion medicine. D-antigen is the most important and highly immunogenic antigen. The anti-D alloantibody causes the hemolytic disease of the newborn and transfusion reaction. Persons are clinically classified as RhD-positive or RhD-negative depending on the presence or absence of D antigen on the red cell surface. The RhD-negative trait can be generated by multiple genetic mechanisms, which have been shown to be ethnic group-dependent. In most RhD-negative Caucasoid individuals, an RhD-negative phenotype is associated with the deletion of RHD gene between the upstream and downstream Rhesus boxes and hybrid Rhesus boxes present on both chromosomes [4, 5]. However, total deletion of RHD gene only accounts for 10%–23% of RhD-negative in African [6] and 60%–70% of RhD-negative in Asian populations [7–10]. In addition, about 15% of Caucasoid people are RhD-negative while in the Asian population, RhD-negative blood type occurs in only 0.1%–0.5% of the population [10–13]. In contrast to RhD-negative Caucasoid persons, approximately 10%–30% of Eastern Asian people who are RhD-negative are DEL phenotype, a rare variant of Rh system in which the D antigen is detectable only by adsorption/elution test [10, 11, 13–15]. 

 The DEL phenotype is serologically designated as a quantitative variant of D antigen. A small amount of anti-D can be eluted from DEL red blood cells (RBCs) after it is incubated with anti-D, although there is no agglutination by the indirect antiglobulin test (IAT). Most DEL donors are typed as RhD-negative because the routine serological typing does not distinguish RhD-negative from the DEL phenotype. Previous reports have indicated that the presence of the *RHD* gene in DEL samples seems to relate strictly to the RhC phenotype with high incidence of RhC(+) in the apparent RhD-negative persons [12, 16, 17]. All DEL persons with intact *RHD* gene showed the CC or Cc phenotype but not the cc phenotype. However, the most frequent cause for DEL phenotype in East Asians is the RHD 1227A allele [7, 13, 18]. In addition, the commercial kit for adsorption/elution test is simple and practical for use in all transfusion patients. Three cases of anti-D immunization by DEL red blood cells have been reported in Austria, Japan, and Korea [19–21]. There is no report about DEL phenotype in Thai RhD-negative blood. Therefore, we investigated the combined performance test of RhC phenotyping, anti-D adsorption/elution test, and RHD 1227A specific sequence primer-polymerase chain reaction (SSP-PCR) for distinguish true RhD-negative from DEL phenotype in the 254 Thai RhD-negative blood donors. The combined test for DEL detection may help to ensure safety in blood transfusion and prevent anti-D alloimmunization without adding much to the costs and time consumption and without incurring an avoidable wastage of RhD-negative blood units that are always not available in Thai and other Asian population.

## 2. Materials and Methods

### 2.1. Blood Samples

 A total of 254 RhD-negative unrelated blood donors were leftover blood samples which were kindly provided by National Blood Centre, The Thai Red Cross Society. The Thai RhD-negative blood samples were used in this study and the other races were excluded with a donor history record. In addition, the blood samples were preserved in citrate phosphate dextrose (CPD) solution and typed as RhD-negative by routine indirect antiglobulin test. All blood samples were tested for RhCE phenotyping and anti-D adsorption/elution test. The RhD-negative samples with RhC(+) were further tested for RHD 1227A polymorphism by specific sequence primer-polymerase chain reaction (SSP-PCR) technique. This research was approved by Thammasat University Ethics Committee.

### 2.2. Serological RhCE Phenotyping

 The 254 RhD-negative blood samples were serotyped for RhC/c and RhE/e antigens by using agglutinating monoclonal anti-C, anti-c, anti-E, and anti-e reagents (DiaClon, DiaMed, Switzerland) in the saline tube test (20 *μ*L of specific monoclonal antibody and 50 *μ*L of 3% red blood cells (RBCs) in saline were added to test tube, centrifuged at 3,400 rpm 15 seconds, and the agglutination was read).

### 2.3. Adsorption/Elution Test

 Adsorption/elution tests were performed on 254 samples. For anti-D adsorption to red blood cells (RBCs), 200 *μ*L of RBCs was incubated for 1 hour at 37°C with 200 *μ*L of monoclonal anti-D IgG (National Blood Centre, The Thai Red Cross Society). The cells were washed and the eluate was prepared by acid elution technique (DiaCidel, DiaMed, Switzerland). The eluates and the last washed supernatants were used for indirect antiglobulin test (IAT) against RhD-positive and RhD-negative cells by using column agglutination technique (LISS/Coombs'card, DiaMed, Switzerland), 25 *μ*L of eluate (or last supernatant), and 50 *μ*L of 1% D positive RBCs in modified LISS (Diluent-II, DiaMed, Switzerland) were added to LISS/Coombs'card, incubated at 37°C 15 minutes, centrifuged at 1,030 rpm for 10 minutes, and the agglutination was read.

### 2.4. Specific Sequence Primer-Polymerase Chain Reaction (SSP-PCR) Analysis for RHD 1227A Allele

 Genomic DNA was extracted by using a commercial kit (AxyPrep Blood Genomic DNA Miniprep Kit, Axygen Biosciences, CA, USA). To screen for the RHD 1227A allele in all samples with RhC(+), the forward primer for RHD 1227A allele: 5′-GATGACCAAGTTTTCTGGAAA-3′, and the reverse primer for RHD 1227A: 5′-GTTCTGTCACCCGCATGTCAG-3′ were used in amplified a 348 bp product. Another pair of nucleotides (the forward primer: 5′-GCCTTCCCAACCATTCCCTTA-3′, the reverse primer: 5′-TAGACGTTGCTGTCAGAGGC-3′) were incubated as an internal control to generate a 629 bp PCR fragment from the growth hormone gene. The PCR reactions were performed at a total volume 10 *μ*L. It contained 1 *μ*L of genomic DNA, 0.5 U DNA polymerase, 200 *μ*M dNTPs, primers, and 2.5 mM MgCl_2_ in the buffer provided by the manufacturer. Forty cycles were programmed on the thermocycler (TGradient, Biometra) as follows: denaturation at 94°C for 5 minutes, then 35 cycles of 30 seconds at 94°C, 40 seconds at 68°C, and 30 seconds at 72°C. PCR products were visualized in a 2% agarose gel.

## 3. Results

As described in Table 1, the 254 RhD-negative samples consisted of 131 ccee, 4 ccEe, 1 ccEE, 101 Ccee, 16 CCee, and 1CcEe. Among them 19.7% (50/254) were typed as DEL phenotype by anti-D adsorption/elution test that consisted of 42 Ccee and 8 CCee. The incidence of RhC(+) of the apparent Thai RhD-negative blood donors was 46.4% (118/254). In addition, the incidence of DEL phenotype in RhC(+) persons as determined by adsorption/elution test was 42.4% (50/118). The distribution of each RhCE phenotype among the 254 RhD-negative samples in this study and the previous report from Taiwan, Hong Kong, and Japan were summarized in Table 2. The incidence of RhD-negative and DEL phenotype were similar among Thai, Taiwanese, and Hong Kong populations. The incidence of true D-negative in Thai population was significantly different from Taiwanese and Hong Kong populations (*P* < 0.05). Statistically, it was significantly different as compared with the Japanese population (*P* < 0.05). However, it is of great interest to note that all DEL persons were highly associated with RhC(+) phenotype in all 4 populations.

**Figure 1:**
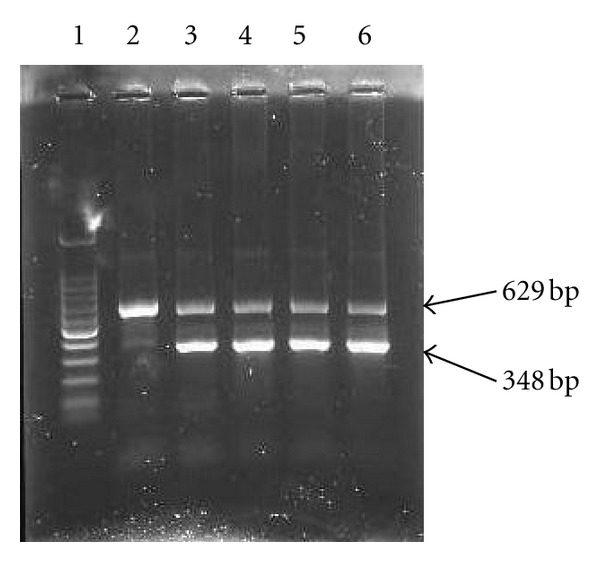
Specific sequence primer-polymerase chain reaction (SSP-PCR) analysis of RHD 1227A polymorphism. The products of SSP-PCR after separation on 2% agarose gel were shown. The specific product were 348 bp, and the internal control product, 629 bp, based on human growth hormone were amplified. Lane 1 = 100 bp ladder marker, lane 2 = true RhD-negative, and lane 3, 4, 5, and 6 = RHD1227A positive.

RHD 1227A polymorphism was detected by SSP-PCR in RhC(+) individuals (Figure 1). The comparative results of SSP-PCR for RHD 1227A and adsorption/elution test in the 118 RhC(+) samples were shown in Table 3. The 48 of the 50 RhD adsorption/elution positive (DEL) samples were also positive for RHD 1227A by SSP-PCR analysis (sensitivity = 96%), and all of RhD adsorption/elution negative samples were negative (specificity = 100%). However, only 2 samples revealed an inconsistent result detected by these two methods. 

## 4. Discussion and Conclusions

DEL phenotype is the weakest known RhD-positive phenotype in the Rh blood group system. A normal RhD-positive red blood cell (RBC) has about 10,000–30,000 D sites per cell depending on the Rh genotypes [22], while the number of D sites on DEL RBC is less than 30 per cell [19]. The incidence of DEL phenotype in Europeans is very low. In contrast, DEL is very common in Eastern Asia such as China, Japan and Korea, and about 10–30% apparently RhD-negative individuals are DEL phenotypes. However, no data has been collected to indicate the frequency of DEL in Africans.

In this study, approximately 20% of apparently Thai RhD-negative blood donors were DEL phenotype by anti-D adsorption/elution test. It is in consistence with the other reports; both Ccee and CCee phenotype were highly prevalent in DEL persons of Taiwanese, Japanese, and Hong Kong populations, whereas the prevalence of the CcEe phenotype was about 44% in DEL group of the Japanese population (Table 2). DEL persons were highly associated with the RhC(+) phenotype in Thai, Taiwanese, Japanese, and Hong Kong populations.

**Figure 2:**
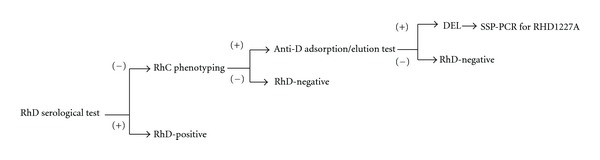
Proposed laboratory protocol for DEL detection.

 For distinguishing the true RhD-negative from DEL phenotype in clinical laboratory, we found that the presence of the intact *RHD* gene in DEL samples was related to the RhC phenotypes with high incidence of RhC(+) in the apparently RhD-negative persons (46.4%), and particularly in DEL persons (100%). All DEL persons with intact *RHD* gene showed the Cc and CC phenotype but not the cc phenotype. Almost the same results were demonstrated in the three Asian populations (Taiwan, Hong Kong, and Japan). In addition, by SSP-PCR analysis, the high sensitivity (96%), and the specificity (100%) were achieved in DEL person for the detection of RHD 1227A, which is a useful genetic marker for DEL identification. DEL red blood cells can induce alloanti-D immunization in the truly RhD-negative recipients. It is important to distinguish true RhD-negative from DEL phenotype in blood donors. As shown in DEL identification test in blood transfusion laboratory, SSP-PCR for RHD 1227A allele is a reliable and simple method for DEL identification in Thai RhD-negative persons and probably in the other Asian populations. We conclude that RhC(+) phenotype, anti-D adsorption/elution test and SSP-PCR are benefit for the identification of RHD 1227A polymorphism. Based on these findings, the combined performance test of RhC phenotyping, anti-D adsorption/elution test, and RHD 1227A specific sequence primer-polymerase chain reaction (SSP-PCR) is useful for distinguishing true RhD-negative from DEL phenotype in the Thai RhD-negative blood donor. As shown in Figure 2, phenotype RhC(+) followed by anti-D adsorption/elution test and RHD 1227A detection can be used as the simple protocol for DEL identification applied in routine laboratory.

## Figures and Tables

**Table 1:** The incidence of RhD-negative, DEL, and true D-negative in Thai population.

Number of apparent Rh phenotypes	254 RhD-negative	50 DEL	204 True D-negative
C (+)			
CCee	16 (6.3%)	8 (16.0%)	8 (3.9%)
CCEe	0	0	0
Ccee	101 (39.7%)	42 (84.0%)	59 (28.9%)
CcEe	1 (0.4%)	0	1 (0.5%)
CcEE	0	0	0
Subtotal	118 (46.4%)	50 (100%)	68 (33.3%)

C (−)			
Ccee	131 (51.6%)	0	131 (64.2%)
ccEe	4 (1.6%)	0	4 (2.0%)
ccEE	1 (0.4%)	0	1 (0.5%)
Subtotal	136 (53.6%)	0	136 (66.7%)

Total	254 (100%)	50 (100%)	204 (100%)

**Table 2:** Distribution of RhD-negative, DEL, and True D-negative phenotypes in four different populations.

No. Apparent Rh phenotypes	This study			Taiwan [17]			Hong Kong [12]			Japan [16]		
	254	50	204	395	126	269	465	136	329	306	102	204
	RhD (−)	DEL	True D (−)	RhD (−)	DEL	True D (−)	RhD (−)	DEL	True D (−)	RhD (−)	DEL	True D (−)
C (+)												
CCee	6.3	16.0	3.9	4.8**	14.3**	0.4*	6.9**	19.1**	1.8**	2.0*	3.92*	0.5*
CCEe	0	0	0	0.3*	0.8	0	0	0	0	0	0	0.5
Ccee	39.7	84.0	28.9	37.0**	83.3**	15.2*	33.8**	78.7**	15.2*	28.0*	52.0*	16.2*
CcEe	0.4	0	0.5	1.3**	1.6**	1.1**	1.1**	2.2**	0.6**	22.0*	44.1*	10.3*
CcEE	0	0	0	0	0	0	0	0	0	0	0	0.5
Subtotal	46.4	100	33.3	43.3	100	16.4	41.7	100	17.6	52.0	100	27.9

C (−)												
ccee	51.6	0	64.2	54.7**	0	80.6*	56.8**	0	80.2*	19.0*	0	28.9*
ccEe	1.6	0	2.0	2.0**	0	3.0**	1.5**	0	2.1**	19.0*	0	28.9*
ccEE	0.4	0	0.5	0	0	0	0	0	0	9.0*	0	14.2*
Subtotal	53.6	0	66.7	56.7	0	83.6	58.3	0	82.3	48.0	0	72.1

*P value < 0.05 = Significantly different.

**P value > 0.05 = Not significantly different.

**Table 3:** Results of SSP-PCR for RHD1227A and adsorption/elution in 118 RhC (+) apparent RhD-negative samples.

RhC (+) (*N* = 118)	RHD1227A (+) (*N* = 50)	RHD1227A (−) (*N* = 68)
Adsorption/elution (+) *N* = 50	48	2
Adsorption/elution (−) *N* = 68	0	68
Sensitivity (%)	96	
Specificity (%)		100
